# Effects of Non-Fermented Red Ginseng Marc in a Commercial Liquid Feeding System on Growth Performance, Fecal Short-Chain Fatty Acids, Blood Profiles, and Pork Quality in Growing Finishing Pigs

**DOI:** 10.3390/ani16111631

**Published:** 2026-05-27

**Authors:** Shenglai Cui, Anran Wu, Yinghai Jin, Xinghao Jin

**Affiliations:** 1 College of Agriculture, Yanbian University, Yanji 133000, China; c18744518961@163.com (S.C.); wuanran324@163.com (A.W.); jinyh@ybu.edu.cn (Y.J.); 2 Institute of Changbai Mountain Specialty Resources, Yanbian University, Yanji 133000, China

**Keywords:** red ginseng marc, liquid feeding, growth performance, nutrient digestibility, growing-finishing pigs

## Abstract

This study evaluates the effects of non-fermented red ginseng marc (RGM) in a commercial liquid feeding system on growth performance, nutrient digestibility, blood profiles, fecal short-chain fatty acids, and pork quality in growing-finishing pigs. A total of 480 crossbred pigs ([Yorkshire × Landrace] × Duroc) with an average body weight of 32.64 ± 0.12 kg were used in a 12-week feeding trial. Experimental pigs were allotted to one of four treatments in a randomized complete block design (RCBD), with three replicate pens per treatment and 40 pigs per pen based on body weight and sex. Dietary red ginseng marc (0, 2%, 3%, 6%) was added to each experimental diet via a liquid feeding system. Final body weight decreased linearly with increasing dietary RGM (*p* < 0.05). Average daily gain during weeks 10–12 showed both linear and quadratic responses (*p* < 0.05), and overall average daily gain during weeks 0–12 decreased linearly (*p* < 0.05). Average daily feed intake decreased linearly during weeks 4–6, 7–9, 10–12, and overall (*p* < 0.05). During weeks 7–9, fecal acetate and butyrate increased linearly (*p* < 0.05), whereas during weeks 10–12, acetate, propionate, butyrate, and total SCFA were reduced at the highest inclusion level. Blood urea nitrogen decreased linearly at measured points (*p* < 0.05). Glucose increased linearly at weeks 9 and 12 (*p* < 0.05), and total cholesterol decreased linearly at week 12 (*p* < 0.05). Under the present commercial liquid feeding conditions, supplemental non-fermented RGM at 2% or 3% of the basal diet could be considered practical inclusion levels, whereas 6% inclusion reduced feed intake and growth performance during the finishing period (weeks 7–12).

## Simple Summary

Red ginseng marc is a residue product generated after red ginseng processing. Although it contains residual nutrients and bioactive compounds, its direct use in pig diets has not been well evaluated, especially under commercial liquid feeding conditions. In this study, non-fermented red ginseng marc was supplemented in liquid diets for growing-finishing pigs at different levels. The inclusion of 2 or 3% showed generally acceptable responses in growth performance, nutrient digestibility and pork quality. However, 6% inclusion reduced feed intake and growth during the finishing period. These results indicate that red ginseng marc can be used in liquid-fed pigs, while high inclusion should be avoided under non-fermented conditions.

## 1. Introduction

The expansion of livestock production has increased the use of feed ingredients such as cereal grains and protein sources [1,2]. Some of these ingredients are also closely linked to human food resources, and the use of agricultural by-products and local feed resources has received increasing attention in pig nutrition [3,4]. In regional pig production, locally available agro-industrial by-products may provide additional feed resources and reduce processing waste. These materials should not be used in pig diets simply because they are locally available, as their composition can vary depending on source, season, and processing conditions [5]. Therefore, their nutrient composition, palatability, and effects on growth performance, digestion, and product quality should be evaluated under practical feeding conditions before they can be recommended [6,7].

Ginseng is an important medicinal crop in Northeast Asia, and Jilin Province is one of the main ginseng-producing areas in China [8]. Red ginseng marc (RGM) is a by-product of red ginseng processing and refers to the solid residue remaining after aqueous extraction and concentration following the washing, steaming, and drying of fresh ginseng [9,10]. Although most water-soluble active compounds are removed during processing, RGM still contains crude protein, fiber fractions, residual ginsenosides, polysaccharides, and mineral components [11]. Previous studies have shown that red ginseng by-products generally contain approximately 4–6% moisture, 13–15% crude protein, 0.8–1.4% crude fat, and 4–5% ash and about 15% crude fiber [12,13]. This composition suggests that RGM may serve as a potential alternative feed ingredient, although its relatively high fiber content may influence nutrient utilization and gut fermentation in monogastric animals.

Most studies on red ginseng and its by-products in monogastric animals have been conducted in poultry [14,15]. Ao et al. [16] reported that supplementation with fermented red ginseng extract in broilers and laying hens did not improve productive performance or egg quality. Similarly, Kim et al. [17] reported that dietary supplementation with 0.5%, 1%, and 2% red ginseng marc in laying hens had limited effects on overall egg production and egg quality, but reduced serum total cholesterol at the 1% and 2% inclusion levels. In broilers, dietary supplementation with red ginseng marc up to 3% did not adversely affect growth performance, while the 3% inclusion level was associated with reduced mortality and serum cholesterol [18]. However, in pigs, studies have mainly focused on fermented red ginseng marc or red ginseng extracts [19,20]. Yin et al. [21] reported that supplementation with fermented red ginseng marc or extract (4 g/kg) had no significant effects on growth performance or blood parameters in weaned piglets. Compared with previous studies, information on the direct use of non-fermented red ginseng marc in pig diets remains scarce, particularly at relatively high inclusion levels.

Commercial liquid feeding systems may provide a practical platform for incorporating by-product ingredients into pig diets because these materials can be easily added and mixed with feed and water. Liquid diets for pigs are generally prepared by mixing feed with water or liquid co-products before delivery through feeding equipment, and previous studies have used or recommended water-to-feed ratios close to 2.6:1 to 3:1 in growing-finishing pigs [22,23]. However, by-products used in liquid feeding systems should be evaluated carefully because their physical properties, palatability, and fermentable components may influence feed intake and hindgut fermentation, especially during prolonged feeding.

Information on the direct use of non-fermented RGM in pig diets is still limited, particularly under commercial liquid feeding conditions. We hypothesized that RGM may be used as a by-product ingredient in liquid-fed growing-finishing pigs, but that its effects would likely depend on the inclusion level. Therefore, this study tested dietary RGM at 0%, 2%, 3%, and 6% to evaluate its suitability in a commercial liquid feeding system and to identify a practical inclusion range based on growth performance, nutrient digestibility, fecal short-chain fatty acids, blood profiles, and pork quality.

## 2. Materials and Methods

### 2.1. Experimental Animals and Management

All experimental procedures were approved by the Yanbian University Institutional Animal Care and Use Committee (YBU-YD2025080001). A total of 480 growing-finishing pigs ([Yorkshire × Landrace] × Duroc; initial body weight, 32.64 ± 0.12 kg) were used in a 12-week feeding trial conducted at a commercial pig farm of Yanbian Hengxing Animal Husbandry Development Co., Ltd. in Longjing, Yanbian, China. Pigs were allotted to a randomized complete block design based on sex and initial body weight, with four dietary treatments, three replicate pens per treatment, and 40 pigs (20 barrows and 20 gilts) per pen. The experimental phases were divided into early growing (weeks 0–3), late growing (weeks 4–6), early finishing (weeks 7–9), and late finishing (weeks 10–12) phases.

All pigs were raised under the routine management conditions of the commercial farm. The experimental houses were cleaned and disinfected before the trial. According to the farm vaccination program, pigs were vaccinated against pseudorabies and classical swine fever at the 2nd and 5th weeks after weaning, and against foot-and-mouth disease at approximately 40 and 60 kg body weight. No in-feed antibiotics were used during the experimental period. Pig health was monitored daily, and the same health management program was applied to all treatments.

### 2.2. Experimental Design and Diets

The dietary treatments were: Control, basal liquid diet without RGM; RGM2, basal liquid diet with 2% RGM; RGM3, basal liquid diet with 3% RGM; and RGM6, basal liquid diet with 6% RGM. The non-fermented RGM used in the present study was provided by Yanbian Kexian Biotechnology Co., Ltd. (Yanbian, China) and was obtained as a by-product after hot-water extraction of red ginseng prepared from 6-year-old Panax ginseng Meyer roots. Fresh ginseng was steamed at 80–90 °C for 3 h and dried at 50–80 °C to produce red ginseng. The red ginseng was then extracted with circulating hot water at 75–90 °C for 8 h, and this extraction process was repeated three times. The remaining solid residue was collected as RGM, dried, and used as the experimental by-product source. Its analyzed chemical composition and ginsenoside profile are shown in Table 1.

A commercial feeding program routinely used on the pig farm was adopted as the basal diet. The commercial basal feed was supplied as mash by Longjing Xinyuan Feed Mill (Yanbian, China). Because the basal diets were proprietary formulations of the feed mill, detailed ingredient composition was not available. The available calculated nutrient composition of the basal diets is provided in Supplementary Table S1. The basal mash feed was first mixed with water at a feed-to-water ratio of 1:3 in a bulk tank to prepare the liquid diet. RGM was then added to the prepared basal liquid diet at different inclusion levels, without replacing any ingredient in the commercial basal feed. The liquid diets were freshly prepared daily and supplied through an automatic computer-controlled liquid feeding system three times daily at 08:00, 15:00, and 23:00 h. The nutrient levels of the basal commercial feed met or exceeded the nutrient requirements suggested by NRC [24]. Preparation and the chemical composition of commercial diets supplemented with red ginseng marc are shown in Table 2.

### 2.3. Growth Performance

Body weight (BW) was measured at the beginning of the experiment and at the end of each phase. Feed intake was recorded on a pen basis throughout the experimental period. Based on BW and feed intake data, average daily gain (ADG), average daily feed intake (ADFI), and gain-to-feed ratio (G:F) were calculated for each phase and for the overall 12-week period.

### 2.4. Nutrient Digestibility and Nitrogen Retention

For the digestibility trial, 16 growing barrows (32.0 ± 0.86 kg) were selected from the same herd as the growth trial. The pigs were assigned to the four dietary treatments, with four pigs per treatment, and were housed individually in metabolic crates. The experimental liquid diets, prepared at a feed to water ratio of 1:3, were fed to pigs 3 times a day at 8:00, 15:00, and 23:00 h. Feed allowance was restricted to approximately two times the maintenance energy requirement, calculated as 106 kcal of ME per kg of BW^0.75^ based on the initial BW of pigs [25]. After 7 days of adaptation, feces and urine samples were collected for 5 days using ferric oxide and chromium oxide as initial and end markers, respectively. Collected excreta were frozen immediately at −20 °C during the collection period, dried (60 °C, 72 h) in an air-drying oven, and ground (5 mm screen, Wiley mill: Thomas Scientific, Swedesboro, NJ, USA) for chemical analysis. Urine samples were also collected daily in a plastic container with 50 mL of 10% H_2_SO_4_ to avoid evaporation of ammonia from urine, and glass wool was used as a filter to remove foreign materials. The collected urine from each pig was brought to a final volume of 4000 mL with water and mixed thoroughly. The representative samples were collected in 50 mL conical tubes and frozen at −20 °C for nitrogen retention analysis.

### 2.5. Sample Collection

At each sampling time, blood and fecal samples were collected from ten pigs per treatment at weeks 3, 6, 9, and 12. The pigs were selected within each treatment based on similar body weight and a comparable sex distribution. Blood was collected from the jugular vein into serum-separation tubes (BD Vacutainer SST™ II Advance; Becton Dickinson, London, UK) and centrifuged at 3000 rpm for 15 min at 4 °C using a refrigerated centrifuge (5810R; Eppendorf, Hamburg, Germany). Serum was transferred into 1.5 mL microtubes and stored at −20 °C until analysis. Fresh fecal samples were collected directly from the rectum of the same pigs, placed into labeled sterile tubes, kept on ice during transfer to the laboratory, and stored at −80 °C for short-chain fatty acid (SCFA) analysis. At the end of the feeding period, pigs remained on their respective diets for an additional 2 weeks until they reached market weight. Thereafter, four pigs per treatment were selected for slaughter based on body weight close to the treatment mean, and longissimus dorsi samples were collected near the 10th rib on the right side of the carcass for meat quality analysis.

### 2.6. Laboratory Analyses

Samples of RGM, experimental diets, feces, urine, and pork were analyzed using standard AOAC procedures [26]. Moisture was determined by oven drying (method 930.15), crude protein by the Kjeldahl method (method 984.13), crude fat as ether extract (method 920.39), crude fiber by the fritted glass crucible method (method 978.10), and ash by incineration (method 942.05). Acid detergent fiber (ADF; method 973.18) and neutral detergent fiber (NDF; method 2002.04) were also determined. Calcium was analyzed by the dry ash method (method 927.02), and total phosphorus was determined using the photometric method (method 965.17). Fecal samples for nutrient analysis were dried in a forced-air oven, ground, and stored in sealed containers before chemical analysis. Urine samples were collected in acidified containers to minimize nitrogen loss, filtered to remove visible impurities, and stored at −80 °C until analysis. Urinary nitrogen was determined by the Kjeldahl method, and nitrogen retention was calculated from nitrogen intake and fecal and urinary nitrogen excretion.

Ginsenosides in RGM were analyzed by high-performance liquid chromatography using an Agilent 1260 Infinity system (Agilent Technologies, Santa Clara, CA, USA). Serum IgG and IgA concentrations were determined using commercial ELISA kits according to the manufacturer’s instructions (Pig IgG ELISA Quantitation Kit and Pig IgA ELISA Quantitation Kit; Bethyl Laboratories, Inc., Montgomery, TX, USA). Serum AST, ALT, BUN, total cholesterol, creatinine, and glucose concentrations were analyzed using an automated blood chemistry analyzer (cobas 8000; Roche Diagnostics, Mannheim, Germany). Fecal short-chain fatty acids (SCFAs), including acetate, propionate, and butyrate, were determined using a Shimadzu GC-2010 Plus gas chromatograph (Shimadzu Corporation, Kyoto, Japan) equipped with a flame ionization detector, as previously described [27].

Meat pH and color were measured at 0, 3, 6, 12, and 24 h postmortem, following commonly used procedures for evaluating pork quality [28]. During the measurement period, pork samples were stored at 4 °C. The pH was determined using a portable pH meter (Model 720; Thermo Orion, Fullerton, CA, USA). Meat color was evaluated based on Commission Internationale de l’Eclairage (CIE) L*, a*, and b* values using a colorimeter (CR-300; Minolta Camera Co., Osaka, Japan). Water-holding capacity (WHC), cooking loss, and shear force were measured as previously described [28]. Briefly, WHC was determined using the centrifugation method. The pork sample was ground, placed in a filter tube, heated in a water bath at 80 °C for 20 min, and centrifuged at 2688× *g* for 10 min at 10 °C using a refrigerated centrifuge. Cooking loss was determined by placing longissimus muscle samples in polyethylene bags and heating them in a water bath until the core temperature reached 72 °C. Samples were weighed before and after cooking, and cooking loss was calculated from the weight difference. After cooking and cooling, samples were cored parallel to the muscle fiber direction using a 0.5-inch-diameter corer, and shear force was measured using a Warner–Bratzler shear device. TBARS values were determined according to the method described by Szuba-Trznadel et al. [29]. Briefly, 3 g of pork sample was homogenized with 9 mL of distilled water and butylated hydroxytoluene solution. The homogenate was reacted with thiobarbituric acid/trichloroacetic acid solution. After heating, cooling, and centrifugation, the absorbance of the supernatant was measured at 532 nm using a spectrophotometer. TBARS values were expressed as mg malondialdehyde/kg meat.

### 2.7. Statistical Analyses

All collected data were analyzed using the General Linear Model procedure of SAS Version 9.4 [30]. Pen was considered the experimental unit for growth performance, whereas the individual pig was considered the experimental unit for digestibility, fecal SCFA, blood profiles, carcass traits, and meat quality measurements. Orthogonal polynomial contrasts were used to evaluate the linear and quadratic responses to increasing dietary RGM supplementation. Because the dietary RGM inclusion levels were not equal, the contrast coefficients were constructed according to the actual inclusion levels of 0, 2, 3, and 6%. Results are presented as means and standard error of the mean (SEM). Statistical significance was declared at *p* < 0.05, and 0.05 ≤ *p* < 0.10 was considered a tendency.

## 3. Results

### 3.1. Growth Performance

The effects of dietary RGM on growth performance are presented in Table 3. The final BW decreased linearly with increasing dietary RGM supplementation (*p* = 0.047). ADG during weeks 10 to 12 showed both linear and quadratic responses (*p* = 0.025 and *p* = 0.027, respectively), and the lowest value was observed in the RGM 6 group. Overall ADG also decreased linearly during weeks 0–12 (*p* = 0.031). ADFI decreased linearly during weeks 4 to 6 (*p* = 0.015), 7 to 9 (*p* = 0.027), 10 to 12 (*p* = 0.015), and 0 to 12 weeks (*p* = 0.046). G:F ratio during weeks 10 to 12 showed a quadratic response (*p* = 0.044).

### 3.2. Nutrient Digestibility and Nitrogen Retention

The effects of dietary RGM on nutrient digestibility and nitrogen retention are presented in Table 4. Dietary RGM supplementation did not affect the apparent total tract digestibility of dry matter, crude protein, crude fat, or crude ash. Nitrogen retention was also not influenced by dietary treatment.

### 3.3. Fecal Short-Chain Fatty Acids (SCFA)

The effects of dietary RGM supplementation on fecal SCFA concentrations are presented in Table 5. At 9 weeks, acetate and butyrate increased linearly with increasing RGM levels (*p* = 0.047 and *p* = 0.036, respectively). At the late finishing stage, acetate, propionate, butyrate, and total SCFA showed linear responses to increasing RGM inclusion (*p* = 0.045, *p* = 0.044, *p* = 0.032, and *p* = 0.042, respectively), with lower values observed in the RGM6 group. In addition, butyrate showed a quadratic response at 12 weeks (*p* = 0.044), while acetate, propionate, and total SCFA showed quadratic tendencies (*p* = 0.056, *p* = 0.081, and *p* = 0.084, respectively).

### 3.4. Blood Profiles

The effects of RGM levels on blood profiles are presented in Table 6. Serum ALT increased linearly at 3, 6, and 12 weeks (*p* = 0.041; *p* = 0.023; *p* = 0.048, respectively). Serum glucose increased linearly at 9 and 12 weeks (*p* = 0.041 and *p* = 0.024, respectively). By contrast, serum BUN decreased linearly with increasing dietary RGM at 3, 6, and 12 weeks (*p* = 0.041, *p* = 0.045, and *p* = 0.043, respectively). Total cholesterol decreased linearly at 12 weeks (*p* = 0.044). No significant effects were observed for AST, creatinine, IgG and IgA concentrations during the whole experimental period.

### 3.5. Proximate Composition and Physicochemical Properties of Pork

The effects of RGM levels on proximate composition and physicochemical properties of pork are presented in Table 7. The moisture, crude protein, crude ash, cooking loss, shear force, water-holding capacity, and TBARS were not affected by treatment.

### 3.6. Meat pH and Color

The effects of dietary RGM on meat pH and color are presented in Table 8 and Table 9. Postmortem pH at 0 h showed a tendency to decrease linearly with increasing RGM levels (*p* = 0.071). However, dietary RGM supplementation did not affect meat color at any measured time.

## 4. Discussion

In the present study, pigs fed 2% or 3% RGM maintained growth performance close to that of the control group, whereas pigs fed 6% RGM showed lower ADG and final BW, especially during the finishing phase. These results suggest that the negative growth response was mainly associated with the high inclusion level during prolonged feeding. Since apparent total tract digestibility and nitrogen retention were not affected, the poorer growth in the 6% group was unlikely to be caused by reduced digestive efficiency. Instead, it was more likely associated with lower daily nutrient intake caused by reduced feed consumption.

The reduction in ADFI may be related to two characteristics of the non-fermented RGM used in this study. First, RGM still contained residual ginsenosides. Pigs are sensitive to taste, generally preferring sweet flavors while avoiding bitter compounds. Ginsenosides such as Rb1, Rg2, Rb3, and Rf are known to contribute to the bitter and astringent taste of ginseng-derived materials [31]. Based on the analyzed total ginsenoside content of RGM and ADFI during weeks 10–12, the estimated total ginsenoside intake was approximately 286.1, 413.5, and 737.3 mg/pig/day in the RGM2, RGM3, and RGM6 groups, respectively. The higher ginsenoside intake in the 6% group may have reduced diet acceptance during prolonged feeding. This interpretation is partly supported by Ao et al. [32], who observed lower ADFI in finishing pigs fed 0.4% fermented red ginseng than in those fed 0.2% during the late finishing period. Moreover, because the RGM used in this study was not fermented, its original bitter and astringent characteristics may have remained in the diet. Fermentation may change the flavor profile of ginseng-derived materials and modify their ginsenoside composition, which could partly weaken unfavorable sensory characteristics [33]. Another possible explanation is that the fiber fraction of RGM may have contributed to the lower ADFI. The analyzed crude fiber content of the diets increased with RGM inclusion. At the 6% inclusion level, this fiber fraction may have increased the bulk or viscosity of the liquid diet after mixing with water. This could have further limited voluntary feed intake during the finishing period. Therefore, the lower ADFI in the 6% group was likely related to both residual bitter compounds and the physical effect of fiber in the liquid diet, leading to lower daily nutrient intake and reduced ADG and final BW.

The present results show that RGM supplementation under liquid feeding conditions did not affect nutrient digestibility or nitrogen retention. This indicates that the lower ADG and final BW in pigs fed 6% RGM were not likely caused by reduced digestive efficiency under liquid feeding. Previous studies have reported that fermented red ginseng or fermented red ginseng marc may improve the apparent total tract digestibility of dry matter or nitrogen in pigs [21,34]. However, these effects are likely associated with fermentation, which can enhance nutrient availability and reduce structural fiber. In the present study, neither the RGM nor the liquid diets were not fermented, which may explain why nutrient digestibility did not improve. Furthermore, Pedersen and Stein [35] reported that liquid feeding at a feed-to-water ratio of 1:3 did not improve the apparent total tract digestibility of DM, GE, or P in growing-finishing pigs, which is consistent with the present results obtained under the same feed-to-water ratio. Overall, RGM supplementation had little effect on digestive efficiency, and the poorer growth performance at 6% RGM was mainly related to lower feed intake.

Dietary supplementation with red ginseng marc altered hindgut fermentation patterns, as reflected by changes in fecal SCFA concentrations. No significant differences were observed during the early growing phase, suggesting that dietary treatment had little effect on hindgut fermentation at this stage. At 9 weeks, acetate and butyrate increased with increasing dietary RGM levels, indicating that moderate inclusion of RGM may provide fermentable substrates for microbial activity. Dietary fiber is known to influence SCFA production depending on its physicochemical properties and fermentability [36], and ginseng-derived polysaccharides have also been reported to modulate intestinal fermentation [37,38]. However, this response did not persist during the late finishing phase. During the late finishing period, acetate and propionate decreased, and butyrate was lowest in pigs fed 6% RGM, suggesting that excessive inclusion may have negative effects on fermentation efficiency under prolonged feeding conditions. Because butyrate is particularly important for intestinal epithelial function and barrier integrity [39], the reduced butyrate concentration in the 6% treatment may reflect a less favorable fermentation profile. These changes in hindgut fermentation coincided with reduced feed intake and poorer growth performance at the highest inclusion level. Therefore, under liquid feeding conditions, inclusion of non-fermented RGM at 2% or 3% may transiently support fermentation, whereas excessive inclusion up to 6% appears to be less favorable for fermentation during the finishing phase.

In the present study, dietary RGM supplementation influenced several blood biochemical parameters, but these changes remained within normal physiological ranges, and no mortality or severe health problems were observed during the experimental period. Serum ALT increased linearly with increasing dietary RGM inclusion, but the values remained within the normal range for pigs (26.0–72.1 U/L) [40], and AST did not show a similar response. The higher intake of residual ginsenosides and other bioactive compounds from non-fermented RGM may have been associated with a mild hepatic metabolic response, although the mechanism remains unclear. This interpretation should be considered cautiously, because ginsenosides are more commonly reported to show hepatoprotective effects in liver injury models, including reductions in serum ALT and AST [41]. Serum BUN decreased with increasing RGM inclusion, which is more likely associated with reduced feed intake and slightly lower daily crude protein intake in the experimental diets. In pigs, BUN is influenced by dietary crude protein concentration, feeding level, and nitrogen intake, and is often used as an indicator of nitrogen utilization and urinary nitrogen excretion [42]. Similarly, the reduction in total cholesterol observed during the late finishing phase may be attributable to decreased energy intake, as lower feed consumption would limit substrate availability for lipid synthesis and deposition [43,44]. In contrast, the increase in serum glucose during the finishing phase remains difficult to interpret. However, serum glucose is a dynamic parameter influenced by feeding program, feeding frequency, and postprandial status in pigs [45], and may reflect variations in feeding behavior under liquid feeding conditions.

In the present study, all pork samples were obtained from pigs slaughtered at a similar body weight (110 kg), thereby minimizing the influence of slaughter weight on meat composition and physicochemical traits. Dietary RGM supplementation did not adversely affect meat quality. Previous studies have reported limited effects of red ginseng or its by-products on meat composition. Park et al. [46] found that 2.5% ginseng by-product did not alter the proximate composition of pork in finishing pigs, although ginsenoside content increased and TBARS values decreased. Similarly, Kim et al. [14] reported that red ginseng marc supplementation at 1%, 2%, and 3% did not affect the proximate composition of broiler meat but reduced TBARS and improved shelf life, likely due to the antioxidant properties of ginsenosides. In contrast, Zhang et al. [34] observed reduced drip loss with low-level inclusion of fermented red ginseng (0.1%) or red ginseng extract (0.1%), without effects on WHC or TBARS. In the present study, ginsenoside deposition in muscle was not determined. Moreover, the unchanged TBARS values suggest that RGM supplementation did not enhance the antioxidant stability of meat.

No significant differences in meat color were observed among treatments, indicating that RGM supplementation had minimal influence on postmortem muscle metabolism. Meat color is influenced by myoglobin status, muscle structure, and light scattering, while pH affects protein denaturation and water-holding capacity [47]. Kim et al. [48] reported that ultimate pH is associated with L*, a*, and b* values as well as muscle composition. Although postmortem pH at 0 h showed a decreasing tendency with increasing RGM inclusion, this response was not maintained from 3 to 24 h. In the present study, the proximate composition of pork was also unchanged, which may partly explain the stable pH and color values.

## 5. Limitations

This study was conducted under a commercial liquid feeding system to evaluate whether non-fermented RGM could be practically used as a locally available by-product feed ingredient for growing-finishing pigs. Under this farm-based setting, the experimental work had to follow the routine feeding and management program of the commercial farm. As a result, the sampling scheme and additional mechanistic measurements were limited. The present study mainly focused on growth performance, nutrient digestibility, blood profiles, fecal SCFA concentrations, and pork quality. Direct measurements of feed preference, liquid diet physical properties, intestinal microbial composition, intestinal morphology, and barrier-related markers were not included. Therefore, the biological mechanisms underlying the responses to RGM inclusion could not be fully clarified. Further studies with mechanistic measurements are needed to explain how non-fermented RGM affects liquid feed characteristics, hindgut fermentation, and intestinal responses, and to provide stronger support for its practical use as a feed resource.

## 6. Conclusions

The present study evaluated the practical inclusion level of non-fermented red ginseng marc (RGM) in growing-finishing pigs under commercial liquid feeding conditions. At 2% and 3% inclusion levels, growth performance values were generally comparable with those of the control group, whereas 6% RGM reduced feed intake and growth performance, particularly during the finishing period. Dietary RGM supplementation had little effect on nutrient digestibility, nitrogen retention, and pork quality, although several blood biochemical parameters changed within physiological ranges. These findings suggest that non-fermented RGM may be used at moderate inclusion levels in commercial liquid-fed pigs, whereas high inclusion should be avoided under non-fermented conditions. The poorer growth response at 6% RGM appeared to be mainly associated with reduced feed intake.

## Figures and Tables

**Table 1:** Analyzed chemical composition and ginsenoside profile of red ginseng marc used in the experiment.

Item	Samples of Red Ginseng Marc ^1^				
	Sample 1	Sample 2	Sample 3	Sample 4	Mean ^2^
**Chemical composition ^3^**					
Dry matter, %	97.39	98.31	98.15	98.16	98.00
Crude protein, % DM	14.15	14.03	13.70	13.69	13.89
Crude fat, % DM	2.21	2.42	2.88	2.88	2.60
Crude fiber, % DM	15.83	16.05	15.89	15.93	15.93
Ash, % DM	2.82	2.90	3.05	3.15	2.98
**Ginsenosides, mg/g**					
Rg1	ND ^4^	ND	ND	ND	ND
Re	ND	ND	ND	ND	ND
Rf	0.16	0.15	0.18	0.25	0.19
Rb1	0.61	0.56	0.57	0.62	0.59
Rg2	0.51	0.54	0.42	0.45	0.48
Rc	ND	ND	ND	ND	ND
Rg6	ND	ND	ND	ND	ND
F4	0.21	0.25	0.31	0.22	0.25
F2	0.53	0.54	0.62	0.45	0.54
Rg3	1.11	1.15	1.04	1.27	1.14
Rk1	1.38	1.21	1.25	1.32	1.29
Rg5	2.34	2.10	2.36	2.15	2.24
**Total ginsenosides**	6.85	6.50	6.75	6.73	6.71

^1^ Samples 1–4 represent four independent red ginseng marc samples collected during the experimental period. ^2^ Values are analyzed values, and the mean was calculated from four samples. ^3^ Dry matter is expressed on an as-fed basis. Crude protein, crude fat, crude fiber, and ash are expressed on a dry matter basis. ^4^ ND, not detected.

**Table 2:** Preparation of experimental liquid diets and chemical composition of basal diet and red ginseng marc mixture before water addition.

A. Preparation of experimental liquid diets ^1^								
**Item**	**Control**		**RGM2**		**RGM3**		**RGM6**	
Commercial basal diet, kg	100		100		100		100	
Water, kg	300		300		300		300	
Basal diet-to-water ratio	1:3		1:3		1:3		1:3	
Supplemental RGM, kg	0		2		3		6	
Final liquid mixture, kg	400		402		403		406	
RGM supplementation level, % of basal diet	0		2		3		6	
RGM proportion in basal diet + RGM mixture, % ^2^	0		1.96		2.91		5.66	
B. Chemical composition of basal diet and RGM mixture before water addition ^3^								
**Item**	**Growing Diet**				**Finishing Diet**			
	**Con**	**R2**	**R3**	**R6**	**Con**	**R2**	**R3**	**R6**
Dry matter, %	91.77	91.80	91.81	91.93	89.93	90.14	90.17	90.34
Crude protein, %DM	19.45	19.37	19.20	19.09	17.62	17.55	17.51	17.40
Crude ash, %DM	4.94	4.90	4.88	4.82	4.88	4.84	4.82	4.86
Crude fat, %DM	3.94	3.92	3.90	3.86	5.02	4.96	4.94	4.87
Crude fiber, %DM	2.94	3.22	3.35	3.74	2.91	3.20	3.35	3.70
ADF, %DM	4.34	5.24	5.67	6.93	4.30	5.22	5.64	6.86
NDF, %DM	11.29	12.19	12.63	13.90	11.73	12.35	13.01	13.87

^1^ The commercial basal diet was first mixed with water at a basal diet-to-water ratio of 1:3, and RGM was then added to the liquid basal diet and did not replace any ingredient in the basal diet. ^2^ RGM proportion in the basal diet + RGM mixture was calculated. ^3^ Dry matter is expressed on an as-fed basis, and crude protein, crude ash, crude fat, crude fiber, ADF, and NDF are expressed on a dry matter basis.

**Table 3:** Effects of supplemental red ginseng marc in a commercial liquid feeding system on growth performance in growing-finishing pigs.

Criteria	Treatment ^1^				SEM ^2^	*p*-Value	
	Control	RGM2	RGM3	RGM6		Linear	Quadratic
**Body weight, kg**							
Initial	32.70	32.70	32.52	32.65	0.311	0.875	0.806
3 wks	46.03	46.18	45.71	45.17	0.455	0.304	0.701
6 wks	60.89	61.08	60.26	59.78	0.471	0.422	0.872
9 wks	76.89	76.90	75.59	73.57	0.619	0.071	0.591
12 wks	92.00	92.39	91.92	86.33	1.060	0.047	0.162
**ADG, g**							
0–3 wks	666.50	673.91	659.54	626.10	12.621	0.238	0.528
4–6 wks	675.57	677.19	661.13	664.14	15.493	0.808	0.944
7–9 wks	761.58	753.32	730.36	656.42	23.742	0.178	0.665
10–12 wks	719.89	737.62	777.29	607.73	26.913	0.025	0.027
0–12 wks	705.99	710.55	707.10	639.05	11.532	0.031	0.127
**ADFI, g/d**							
0–3 wks	1474.40	1469.40	1455.49	1488.74	21.903	0.783	0.624
4–6 wks	1885.34	1907.97	1826.19	1850.25	10.671	0.015	0.246
7–9 wks	2080.30	2018.62	1947.19	1913.52	25.992	0.027	0.395
10–12 wks	2161.91	2174.09	2115.55	1941.29	33.283	0.015	0.114
0–12 wks	1900.49	1892.52	1836.11	1798.45	17.346	0.046	0.854
**G:F ratio**							
0–3 wks	0.452	0.459	0.454	0.420	0.005	0.152	0.105
4–6 wks	0.358	0.355	0.362	0.359	0.009	0.932	0.994
7–9 wks	0.366	0.374	0.375	0.345	0.011	0.534	0.525
10–12 wks	0.333	0.338	0.368	0.313	0.010	0.265	0.044
0–12 wks	0.372	0.375	0.385	0.355	0.004	0.184	0.095

^1^ Control, basal diet; RGM2, basal diet + 2% red ginseng marc; RGM3, basal diet + 3% red ginseng marc; RGM6, basal diet + 6% red ginseng marc. ^2^ SEM, standard error of the mean.

**Table 4:** Effects of supplemental red ginseng marc on apparent total tract digestibility and nitrogen retention in growing pigs ^1^.

Criteria	Treatment ^2^				SEM ^3^	*p*-Value	
	Control	RGM2	RGM3	RGM6		Linear	Quadratic
Dry matter, %	82.18	82.36	82.54	81.88	0.442	0.412	0.345
Crude protein, %	79.42	79.67	79.81	79.06	0.493	0.372	0.286
Crude fat, %	67.51	68.14	67.93	66.97	0.815	0.393	0.374
Crude ash, %	44.73	44.86	44.18	44.95	0.646	0.367	0.267
Nitrogen retention, %	57.42	56.18	56.94	55.37	0.921	0.216	0.192

^1^ A total of 16 crossbred growing barrows with an average initial body weight of 32.0 ± 0.86 kg were individually allotted to four dietary treatments, with four pigs per treatment. ^2^ Control, basal diet; RGM2, basal diet + 2% red ginseng marc; RGM3, basal diet + 3% red ginseng marc; RGM6, basal diet + 6% red ginseng marc. ^3^ SEM, standard error of the mean.

**Table 5:** Effects of supplemental red ginseng marc in a commercial liquid feeding system on fecal short chain fatty acids in growing-finishing pigs.

Criteria	Treatment ^1^				SEM ^2^	*p*-Value	
	Control	RGM2	RGM3	RGM6		Linear	Quadratic
**Acetate, ** **µ** **mol/g**							
Initial	39.1						
3 wks	42.50	43.00	42.80	42.20	1.20	0.584	0.621
6 wks	43.00	45.50	46.00	45.20	1.40	0.095	0.212
9 wks	44.20	46.00	46.50	46.50	1.50	0.047	0.173
12 wks	44.00	45.80	45.20	40.50	1.51	0.045	0.056
**Propionate, ** **µ** **mol/g**							
Initial	13.6						
3 wks	14.50	14.80	14.60	14.20	0.45	0.475	0.556
6 wks	14.80	15.80	16.00	15.60	0.60	0.216	0.238
9 wks	15.20	16.00	16.20	15.40	0.70	0.082	0.197
12 wks	15.80	16.20	16.00	14.20	0.65	0.044	0.081
**Butyrate, ** **µ** **mol/g**							
Initial	11.4						
3 wks	10.20	10.30	11.10	10.20	0.20	0.622	0.554
6 wks	10.50	11.20	11.30	11.10	0.24	0.082	0.214
9 wks	10.80	11.00	11.10	10.50	0.22	0.036	0.185
12 wks	10.60	10.90	10.80	9.60	0.30	0.032	0.044
**Total SCFA, ** **µ** **mol/g**							
Initial	65.32						
3 wks	67.50	68.20	67.80	67.00	1.76	0.484	0.601
6 wks	72.00	72.50	73.00	72.00	1.87	0.122	0.194
9 wks	71.00	73.50	74.00	69.50	1.56	0.074	0.155
12 wks	70.50	73.00	72.50	69.50	1.34	0.042	0.084

^1^ Control, basal diet; RGM2, basal diet + 2% red ginseng marc; RGM3, basal diet + 3% red ginseng marc; RGM6, basal diet + 6% red ginseng marc. ^2^ SEM, standard error of the mean.

**Table 6:** Effects of supplemental red ginseng marc in a commercial liquid feeding system on blood profiles in growing-finishing pigs.

Criteria	Treatment ^1^				SEM ^2^	*p*-Value	
	Control	RGM2	RGM3	RGM6		Linear	Quadratic
**ALT ^3^, IU/L**							
3 wks	30.37	34.25	33.25	36.50	0.931	0.041	0.714
6 wks	32.37	31.87	33.13	32.50	1.863	0.023	0.505
9 wks	29.62	32.88	31.13	32.50	0.771	0.312	0.542
12 wks	31.63	33.00	32.37	35.75	1.223	0.048	0.421
**AST, IU/L**							
3 wks	32.25	30.25	34.25	29.12	1.606	0.922	0.931
6 wks	33.87	36.25	31.25	29.50	4.505	0.851	0.992
9 wks	33.62	33.37	33.62	32.50	1.549	0.674	0.793
12 wks	27.25	27.50	27.87	25.62	1.223	0.705	0.965
**BUN, IU/L**							
3 wks	9.95	8.95	8.93	7.52	0.375	0.041	0.956
6 wks	10.38	9.38	9.45	8.15	0.340	0.045	0.985
9 wks	11.06	10.06	9.83	8.70	0.470	0.061	0.881
12 wks	12.27	11.27	11.11	9.86	0.371	0.043	0.892
**Creatinine, mg/dL**							
3 wks	1.16	1.08	1.13	1.23	0.025	0.224	0.121
6 wks	1.26	1.18	1.24	1.38	0.293	0.202	0.112
9 wks	1.80	1.31	1.35	1.58	0.103	0.602	0.084
12 wks	1.51	1.43	1.48	1.63	0.038	0.191	0.176
**Glucose, mg/dL**							
3 wks	89.12	88.13	88.63	88.14	0.966	0.765	0.892
6 wks	95.87	96.12	97.75	100.13	1.393	0.254	0.803
9 wks	89.25	92.25	90.12	103.5	3.066	0.041	0.434
12 wks	76.37	83.37	88.63	89.50	2.034	0.024	0.255
**Total Cholesterol, mg/dL**							
3 wks	90.37	90.55	90.85	89.93	1.303	0.893	0.846
6 wks	88.50	91.00	92.00	90.75	1.653	0.692	0.585
9 wks	91.63	91.41	88.75	87.90	0.869	0.103	0.931
12 wks	93.75	93.88	87.25	87.23	0.873	0.044	0.212
**IgG, mg/mL**							
3 wks	9.85	9.85	9.43	9.84	0.091	0.841	0.155
6 wks	9.45	9.81	9.78	9.32	0.111	0.482	0.127
9 wks	9.85	9.39	9.64	9.12	0.138	0.122	0.987
12 wks	9.67	9.70	9.63	9.85	0.134	0.273	0.454
**IgA, mg/mL**							
3 wks	1.70	2.29	2.36	1.96	0.168	0.772	0.177
6 wks	1.77	1.61	1.19	2.42	0.202	0.202	0.091
9 wks	1.41	1.48	1.63	1.66	0.128	0.466	0.595
12 wks	1.95	1.50	1.60	1.49	0.052	0.385	0.544

^1^ Control, basal diet; RGM2, basal diet + 2% red ginseng marc; RGM3, basal diet + 3% red ginseng marc; RGM6, basal diet + 6% red ginseng marc. ^2^ SEM, standard error of the mean. ^3^ ALT, alanine aminotransferase; AST, aspartate aminotransferase; BUN, blood urea nitrogen; IgG, immunoglobulin G; IgA, immunoglobulin A.

**Table 7:** Effects of supplemental red ginseng marc in a commercial liquid feeding system on proximate composition and physicochemical properties of pork in growing-finishing pigs.

Criteria	Treatment ^1^				SEM ^2^	*p*-Value	
	Control	RGM2	RGM3	RGM6		Linear	Quadratic
**Proximate composition, %**							
Moisture	75.92	74.85	75.04	74.36	0.515	0.322	0.781
Crude protein	27.64	26.41	26.95	25.88	0.663	0.443	0.912
Crude fat	6.41	6.14	6.43	6.29	0.372	0.412	0.155
Crude ash	0.51	0.48	0.44	0.57	0.084	0.771	0.632
**Physicochemical properties**							
Cooking loss, %	27.74	26.50	27.05	25.98	0.663	0.442	0.914
Shear force (kg/0.5 inch^2^)	47.83	44.90	48.68	46.19	2.901	0.924	0.992
WHC, %	64.43	66.45	64.22	64.74	0.946	0.942	0.774
TBARS	0.067	0.064	0.068	0.066	0.008	0.724	0.904

^1^ Control, basal diet; RGM2, basal diet + 2% red ginseng marc; RGM3, basal diet + 3% red ginseng marc; RGM6, basal diet + 6% red ginseng marc. ^2^ SEM, standard error of the mean. WHC, water-holding capacity; TBARS, thiobarbituric acid reactive substances.

**Table 8:** Effects of supplemental red ginseng marc in a commercial liquid feeding system on pH of pork in growing-finishing pigs.

Criteria	Treatment ^1^				SEM ^2^	*p*-Value	
	Control	RGM2	RGM3	RGM6		Linear	Quadratic
**pH**							
0 h	6.11	5.95	5.67	5.66	0.080	0.071	0.174
3 h	5.61	5.61	5.48	5.62	0.058	0.952	0.592
6 h	5.49	5.77	5.50	5.68	0.059	0.446	0.576
12 h	5.55	5.77	5.62	5.80	0.053	0.185	0.814
24 h	5.66	5.72	5.78	5.74	0.051	0.694	0.632

^1^ Control, basal diet; RGM2, basal diet + 2% red ginseng marc; RGM3, basal diet + 3% red ginseng marc; RGM6, basal diet + 6% red ginseng marc. ^2^ SEM, standard error of the mean.

**Table 9:** Effects of supplemental red ginseng marc in a commercial liquid feeding system on meat color of pork in growing-finishing pigs.

Criteria	Treatment ^1^				SEM ^2^	*p*-Value	
	Control	RGM2	RGM3	RGM6		Linear	Quadratic
**CIE L** *							
0 h	36.51	35.35	32.80	35.61	1.591	0.714	0.351
3 h	39.46	36.15	38.41	40.05	1.718	0.625	0.143
6 h	43.15	43.30	43.11	38.20	1.995	0.194	0.462
12 h	36.43	32.65	34.13	32.64	1.784	0.121	0.135
24 h	38.62	34.52	36.28	37.47	1.723	0.272	0.146
**CIE a** *							
0 h	6.06	5.73	6.60	6.98	0.551	0.214	0.734
3 h	4.37	4.55	4.03	4.56	0.248	0.225	0.385
6 h	6.00	6.03	6.32	6.29	0.168	0.746	0.776
12 h	5.14	7.11	4.99	3.59	0.142	0.224	0.367
24 h	6.10	4.41	6.22	4.13	1.098	0.235	0.281
**CIE b** *							
0 h	8.16	9.12	9.19	8.93	0.473	0.341	0.212
3 h	11.01	11.18	9.50	10.18	0.779	0.423	0.423
6 h	10.32	11.64	11.01	11.69	0.643	0.192	0.254
12 h	10.40	11.95	10.04	9.91	0.940	0.464	0.574
24 h	11.15	9.85	10.20	9.37	0.753	0.425	0.812

^1^ Control, basal diet; RGM2, basal diet + 2% red ginseng marc; RGM3, basal diet + 3% red ginseng marc; RGM6, basal diet + 6% red ginseng marc. ^2^ SEM, standard error of the mean.

## Data Availability

Upon reasonable request, the datasets of this study can be made available from the corresponding author.

## Supplementary Materials

The following supporting information can be downloaded at https://www.mdpi.com/article/10.3390/ani16111631/s1. Table S1. Calculated nutrient composition of commercial basal diets used in the growing and finishing phases.

## Abbreviations

The following abbreviations are used in this manuscript:

RGM	Red ginseng marc
RCBD	Randomized complete block design
BUN	Blood urea nitrogen
NRC	National Research Council
BW	Body weight
ADG	Average daily gain
ADFI	Average daily feed intake
G:F ratio	Gain:feed ratio
SCFA	Short-chain fatty acid
ME	Metabolizable energy
AOAC	Association of official analytical chemists
ADF	Acid detergent fiber
NDF	Neutral detergent fiber
IgG	Immunoglobulin G
IgA	Immunoglobulin A
AST	Aspartate aminotransferase
ALT	Alanine aminotransferase
FID	Flame ionization detector
CIE	Commission internationale de l’eclairage
WHC	Water-holding capacity
SEM	Standard error of the mean
