# Supplementary material for: Effects of Non-Fermented Red Ginseng Marc in a Commercial Liquid Feeding System on Growth Performance, Fecal Short-Chain Fatty Acids, Blood Profiles, and Pork Quality in Growing Finishing Pigs
Source: Animals (Basel). 2026 May 27;16(11):1631. doi: 10.3390/ani16111631 (PMC13255600; doi:10.3390/ani16111631)
Supplement: Supplementary file 1 [file animals-16-01631-s001.zip › animals-4300376-supplementary.pdf]

Table S1. Calculated nutrient composition of commercial basal diets used in the growing and finishing phases[1]

Item	Growing basal diet	Finishing basal diet
ME, kcal/kg	3350.00	3350.00
Crude protein, %	18.00	16.00
Crude fiber, %	2.75	2.72
Total calcium, %	0.67	0.52
Total phosphorus, %	0.56	0.47
SID lysine, %	0.85	0.70
SID methionine, %	0.26	0.23
SID threonine, %	0.55	0.51
SID tryptophan, %	0.17	0.15
SID valine, %	0.96	0.66

[1)] Quantities of vitamins and minerals provided per kg of commercial feed (growing and finishing diets): vitamin A, 8,000 IU; vitamin D3, 800 IU; vitamin E, 40 mg; vitamin. K3, 4 mg; vitamin B1, 2 mg; vitamin B2, 9.2 mg; vitamin B6, 3 mg; calcium pantothenic acid, 20 mg; niacin, 50 mg; Folic acid, 600 ug; D-biotin, 200 ug; vitamin B12, 30 ug; Fe, 80 mg; Cu, 20 mg; Zn, 60 mg; Mn, 40 mg; I, 0.45 mg; Se, 0.15 mg; Co, 0.5 mg.

ME, metabolizable energy; SID, standardized ileal digestibility.
